# Post-Stroke Pneumonia in Real-World Practice: Background, Microbiological Examination, and Treatment

**DOI:** 10.3390/neurolint15010006

**Published:** 2023-01-09

**Authors:** Takayoshi Akimoto, Makoto Hara, Masaki Ishihara, Katsuhiko Ogawa, Hideto Nakajima

**Affiliations:** Division of Neurology, Department of Medicine, Nihon University School of Medicine, Itabashi-ku, Tokyo 173-8610, Japan

**Keywords:** acute ischemic stroke, sputum, prognostic, pneumonia, nasogastric tubes

## Abstract

Post-stroke pneumonia (PSP) has an impact on acute ischemic stroke (AIS). Although predictive scores for PSP have been developed, it is occasionally difficult to predict. Clarifying how PSP was treated after its onset in clinical practice is important. Admitted patients with AIS over a 2-year period were retrospectively reviewed. Of 281 patients with AIS, 24 (8.5%) developed PSP. The integer-based pneumonia risk score was higher in patients with PSP. The onset of PSP was frequently seen up to the 4th day of hospitalization. Of patients with PSP, sputum examination yielded Geckler 4 or 5 in only 8.3%. Angiotensin-converting enzyme inhibitor (ACE-I) was more frequently administered to patients with PSP; however, all these cases were started with ACE-I following PSP onset. Nasogastric tubes (NGTs) were inserted in 16 of the patients with PSP, of whom 11 were inserted following PSP onset. Multivariate analysis showed that PSP onset was a poor prognostic factor independent of the female sex, urinary tract infection, and National Institutes of Health Stroke Scale. PSP treatment would benefit from the administration of antimicrobials and ACE-I, as well as NGT insertion. To select effective agents for PSP and evaluate the indications for NGT insertion, further case studies are needed.

## 1. Introduction

Pneumonia often occurs during the course of stroke and is termed post-stroke pneumonia (PSP). The incidence of PSP is approximately 3.53–31.3%, depending on patient background and underlying datasets [1,2,3,4,5,6,7,8,9,10]. PSP affects stroke outcomes [1,11,12], length of hospital stay [1,11], and healthcare costs [13]. Therefore, to improve patient outcomes, constant assessment of PSP in stroke care is needed. Several risk factors and predictive scores for PSP onset have been investigated [3,5,7,8,9,10]. Preventive antimicrobial therapy did not improve PSP outcomes [14,15], and there has not been a defined method for improving PSP outcomes. Therefore, the establishment of evaluation criteria and therapeutic measures for PSP is an important issue for stroke care in a more aged population. This study aimed to examine the risk factors for PSP and the impact on prognosis, and discusses strategies for PSP to improve outcomes.

## 2. Materials and Methods

### 2.1. Patient Collection and Definition of Acute Ischemic Stroke (AIS)

Patients admitted to our department from 26 October 2015 to 26 October 2017 with a diagnosis of AIS were retrospectively reviewed from our database. In this study, AIS was defined as patients presenting with acute-onset (1–7 days) neurological symptoms and high-intensity areas on diffusion-weighted magnetic resonance imaging or low-density areas on computed tomography in areas consistent with neurological symptoms. Patients with no lesions on initial imaging studies and lesions on reexamination were included in AIS.

### 2.2. Definition of PSP

To diagnose PSP, we referred to the diagnostic criteria of “probable SAP” in “Recommended Diagnostic Criteria for Definite and Probable SAP in Patients Not Receiving Mechanical Ventilation” by the Stroke Consensus Group [16]. In this diagnostic criteria, PSP diagnosis requires one of the following three: (1) fever (>38 °C); (2) leukopenia or leukocytosis (white blood cells [WBC] < 4000 or > 12,000/mm^3^); or (3) alteration of unexplained state of consciousness, and two of the following four: (1) increased airway secretions; (2) clinical symptoms, including cough or dyspnea; (3) worsening respiratory sounds on auscultation; and (4) hypoxemia [16]. This criteria recommended using the term “hospital-acquired pneumonia” for pneumonia that develops for more than 7 days after the onset of AIS [16]. However, we found patients whose neurological symptoms worsened after admission and who developed pneumonia 7 days after admission. We included these cases; therefore, we defined PSP as all cases wherein pneumonia developed during admission for AIS in the present study.

### 2.3. Collected Data

We collected the following patients’ information: age, sex, independent or not before stroke onset, past medical history (e.g., hypertension, diabetes mellitus, and stroke), smoking habit, neurological findings on admission (e.g., Glasgow coma scale [GCS], hemiparesis, dysarthria, facial weakness, and National Institutes of Health Stroke Scale [NIHSS]), subtypes of ischemic stroke based on the TOAST classification (large-artery atherosclerosis [LA], cardioembolism [CE], small-vessel occlusion [SV], stroke of other determined etiology, two or more causes identified, or negative evaluation) [17], the location of the lesion (supratentorial, infratentorial), complication of urinary tract infection (UTI), prescription at discharge (e.g., cilostazol and angiotensin-converting enzyme inhibitor [ACE-I]), outcome at discharge (e.g., duration of admission and modified Rankin scale [mRS]), and meal at discharge (e.g., normal meal, soft meal, tube feeding, or intravenous hyperalimentation). We considered mRS 0–2 and 3–6 as good and poor outcomes at discharge, respectively. No patients received t-PA and endovascular treatment.

We calculated the integer-based pneumonia risk (ISAN) score, which was a prognostic score for PSP, and assessed by age (<60, 60–69, 70–79, 80–89, and >90 years as 0, 3, 4, 6, and 8 points, respectively), sex (female, 0; male, 2 points), NIHSS score on admission (0–4, 5–15, 16–20, and >21 as 0, 4, 8, and 10 points, respectively), and pre-independence (independent, 0; not independent, 2 points) [10]. Based on the total ISAN score, the risk of PSP was classified into the following four groups: 0–5, 6–10, 11–14, and >15 points as low-, medium-, high-, and very high-risk groups, respectively [10].

### 2.4. Microbiological Examination

In the case of PSP, all patients underwent microbiological examination of sputum. The sputum was collected on sterile sputum containers by patient self-expectoration or aspiration, and the results were reported according to the Geckler group classification in a bacteriology laboratory. In the Geckler classification, a laboratory technician counts the number of buccal squamous epithelial (BSE) cells and WBCs in the 100× view field [18]. Bacterium cultured from the sputum of group 5 (BSE cells < 10/field; WBC > 25/field) or 4 (BSE cells < 10–15/field; WBC > 25/field) was considered a good-quality specimen. Since the bacteria cultured from group 3 (BSE cells > 25/field; WBC > 25/field) specimens were also possible causative agents [18], we examined cases in groups 3–5. For cases with repeated PSP, the first specimen was investigated.

### 2.5. Statistical Analysis

Statistical analysis was performed using SPSS 28.0 (IBM Corporation, Tokyo, Japan), PSP and non-PSP groups, and good and poor outcome groups, respectively. The Fisher exact probability test was used for the nominal scale, and the Mann–Whitney U test for the ordinal and continuous scales. Variation in three or more groups was tested using the Kruskal–Wallis test. To determine whether PSP was an independent poor prognostic factor, logistic regression analysis with stepwise (maximum likelihood) increasing variables was performed, with items that were expected to be less confounding as independent variables and the good/poor outcome group as dependent variables. All *p* values < 0.05 were considered statistically significant.

## 3. Results

### 3.1. PSP versus Non-PSP

During the analysis period, 302 patients with AIS were admitted. Among these patients, 21 were diagnosed with transient ischemic attack and were subsequently excluded. Of the remaining 281 patients (72.4% male, median age 73 years), 24 (8.5%) developed PSP. The results of the univariate analysis for PSP and non-PSP are presented in Table 1. No significant difference was noted between the PSP and non-PSP groups in age and sex; however, the degree of independence before illness was higher in the non-PSP group (*p* = 0.021; PSP: 70.8% vs. non-PSP: 88.7%). Regarding medical history, the PSP group had a significantly higher rate of hypertension (*p* = 0.045; 83.3% vs. 62.3%), whereas diabetes and stroke showed no significant difference. Furthermore, smoking habit was not significant. In the PSP group, neurological findings on admission were lower GCS score (*p* < 0.001; median: 14 vs. 15 points), higher rate of dysarthria (*p* = 0.049; 58.3% vs. 37.0%), and higher NIHSS score (*p* = 0.007; 4 vs. 2 points); however, rates of hemiparesis and facial weakness were not significant. The cause of AIS was identified in 189 of 281 cases (67.3%). Of the identified causes, CE was the most common (73/281 cases, 26.5%). The variation in stroke subtype between the PSP and non-PSP groups was not significant. In the PSP group, a higher rate of CE (PSP vs. non-PSP: 33.3% vs. 25.3%) and a lower rate of ATIS (4.2% vs. 11.7%) and SV (8.3% vs. 24.1%) were observed in each subtype. In the PSP group, there were significantly more patients with infratentorial lesions (*p* = 0.042, 41.7% vs. 23.0%), and there was no significant difference in patients with supratentorial lesions. The ISAN score was significantly higher in the PSP group (*p* < 0.001; 8 vs. 5 points). Moreover, the risk grouping showed that low risk had a lower rate in the PSP group (37.5% vs. 60.7%), medium risk was similar in both groups (29.2% vs. 29.2%), and high risk (29.2% vs. 8.6%) and very high risk (4.2% vs. 1.6%) had higher rates in the PSP group. The rate of cilostazol prescription was not significantly different at discharge. ACE-I was administered more frequently in the PSP group (*p* = 0.046; 16.7% vs. 5.1%); however, it was started following the onset of PSP in all patients who received it. Patients with PSP were more likely to present with a poor outcome at discharge (*p* < 0.001; 75.0% vs. 30.3%). Only 19.0% of the patients with PSP were on a regular meal at discharge. Nasogastric tube (NGT) feeding was received by 33.3% and 4.7% of the patients in the PSP and non-PSP groups at discharge, respectively.

### 3.2. Details of PSP Cases

**Figure 1:**
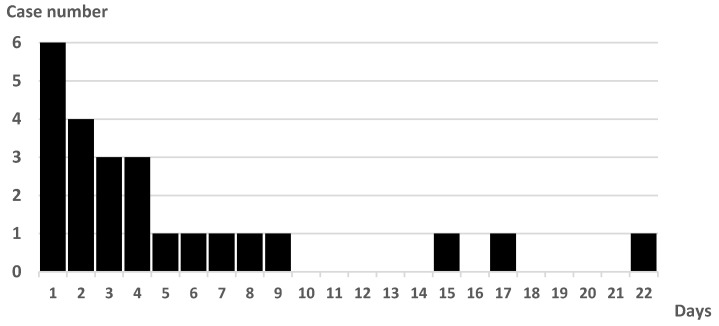
Number of cases and days from admission to PSP onset.

**Figure 2:**
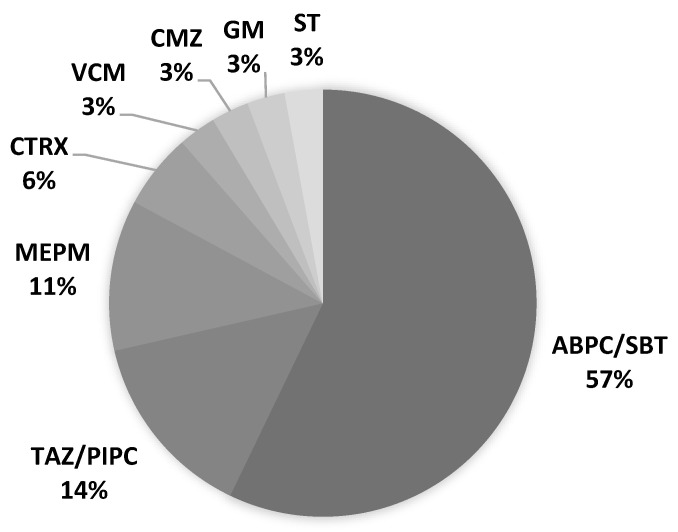
Antimicrobial agents selected for PSP treatment. ABPC/SBT: ampicillin/sulbactam, CMZ: cefmetazole, CTRX: ceftriaxone, GM: gentamicin, MEPM: meropenem, ST: sulfamethoxazole/trimethoprim, TAZ/PIPC: tazobactam/piperacillin, VCM: vancomycin.

The onset of PSP was 67% on days 1–4 after admission (day 1: day of admission). The latest onset of PSP from admission was on day 22 (Figure 1). All patients had sputum cultures before antimicrobial administration. The initial culture examination revealed 1, 2, 17, and 4 cases of Geckler 5, 4, 3, and 2 or less, respectively. Enterobacter aerogenes was detected in the sputum of Geckler 5 methicillin-sensitive Staphylococcus aureus (MSSA), and Klebsiella pneumoniae (K. pneumoniae) and Streptococcus agalactiae were detected in the sputum of Geckler 4. MSSA, K. pneumoniae, Serratia marcescens, and Stenotrophomonas maltophilia were cultured in relatively high numbers from the sputum of Geckler 3. Culture results are shown in Table S1. Ampicillin/sulbactam (ABPC/SBT) was the most frequently selected antimicrobial (57%) (Figure 2). Among the 24 patients with PSP, 8 had no NGT insertion, 5 had NGT insertion before the onset of PSP, and 11 had NGT insertion after the onset of PSP. Seven patients had NGTs inserted at discharge. Three deaths occurred in the PSP group. The cause of death was cerebral herniation due to worsening cerebral infarction in 2 patients, and the combination of PSP and candidiasis in 1 patient.

### 3.3. Good versus Poor Outcome

The poor outcome group (mRS ≥ 3 at discharge) was significantly older (*p* < 0.001; good vs. poor outcome: median, 71 vs. 79 years), had more female patients (*p* = 0.002; 26.6% vs. 44.3%), and was less pre-independent before admission (*p* < 0.001; 100% vs. 62.9%), with higher GCS (*p* < 0.001; 15 vs. 15 points) and NIHSS scores (*p* < 0.001; 1 vs. 4 points) and higher rates of hemiparesis (*p* < 0.001; 38.6% vs. 76.3%), dysarthria (*p* = 0.011; 33.7% vs. 48.5%), and facial weakness (*p* = 0.007; 19.0% vs. 33.0%). A significant variation in stroke subtype was noted between the two groups (*p* = 0.002). In the poor outcome group, LA (good vs. poor outcome: 7.6% vs. 17.5%) and CE (24.5% vs. 28.9%) were relatively more common, and SV (28.3% vs. 12.4%) was less common. UTI complications were higher in the poor prognosis group (*p* < 0.001; 4.9% vs. 21.6%). Moreover, significant differences were noted in the length of hospital stay (*p* < 0.001; 16 vs. 37 days) and percentage of oral intake at discharge (*p* < 0.001) (Table 2).

Multivariate analysis was performed to determine whether PSP was an independent poor prognostic factor, with age, sex, NIHSS, UTI, and PSP as independent variables and good/poor outcome as the dependent variable, and it was found that PSP (aOR, 6.903; 95% confidence interval [CI], 2.080–22.915), NIHSS (aOR, 1.885; 95% CI, 1.563–2.273), and sex (female) (aOR, 2.876; 95% CI, 1.473–5.613) were independent poor outcome factors (Table 3). In this model, the sensitivity was 91.9%, specificity was 61.5%, positive predictive value was 82.1%, negative predictive value was 79.7%, and diagnostic accuracy was 81.5%.

## 4. Discussion

We investigated the background, incidence, pathogenesis, and treatment of PSP in patients hospitalized for AIS. Pre-stroke independence, history of hypertension, low GCS score, high NIHSS score, and dysarthria were risk factors for PSP. The ISAN score, a predictive score for PSP [10], was significantly higher in the PSP group than in the non-PSP group; 62.5% of the patients with PSP were rated as medium-risk or higher using the ISAN score risk classification. The sputum cultures of patients with PSP showed that 8.3% were Geckler 4 or 5. The treatment of PSP would benefit from the administration of antimicrobials, such as ABPC/SBT, and NGT insertion. ACE-I was administered more frequently in the PSP group; however, all these cases were started with ACE-I following PSP onset. PSP was a poor prognostic factor for stroke independent of age, sex, and NIHSS.

Some studies have identified older age [3,5,8,9] and sex (male) [3,8,10] as background factors for PSP; however, in the present study, no significant differences in these factors were observed between the PSP and non-PSP groups. In the PSP group, more patients had a history of hypertension. Although there are reports that PSP should be noted when SBP is >200 mmHg after admission [5], there were no reports of hypertension being a risk factor for PSP. Neurological findings included low GCS and high NIHSS scores, as well as dysarthria, which was more common in the PSP group. A correlation between dysphagia and severe dysarthria caused by damage to the glossopharyngeal nerve has been reported in patients with brainstem stroke [19]. This study did not assess for dysphagia; however, it suggests that patients with dysarthria had concomitant dysphagia. The PSP group had a slightly higher proportion of CE as a cause of AIS; however, no significant deviation in the stroke subtype was observed between the PSP and non-PSP groups. Although it has been reported that the stroke subtype was related to the cause of PSP [7], it is necessary to analyze more cases to determine the relationship between the stroke subtype and PSP development. In addition to PSP, vascular and urinary complications have been shown to be a risk for long-term stay in stroke patients [20]. In our study, PSP, but not UTI, was shown to be an independent poor prognostic factor. We attribute this result to the fact that UTIs are treated by administering antimicrobials and reducing the use of urinary catheters, whereas PSP requires focused intervention on nutritional intake methods in addition to antimicrobial therapy.

Since using a single indicator for predicting the onset of PSP is challenging, several studies have been conducted to combine multiple indicators [3,5,7,8,9,10]. Although several of these indicators have included dysphasia as a predictor [3,8,9], there has been no established method for evaluating the presence or absence of dysphagia in patients with stroke [21,22]. Therefore, the ISAN score is a PSP predictive score developed without the inclusion of dysphagia [10]. In the present study, the PSP group had a significantly higher ISAN score than the non-PSP group. However, 37.5% of the PSP group was assessed as ISAN low-risk, and 39.3% of the non-PSP group was assessed as ISAN medium-risk or higher, suggesting that it was difficult to accurately predict PSP.

We examined the details of the PSP cases. In the present study, PSP onset occurred most frequently up to the fourth day of admission. There were five cases of PSP after the eighth day of admission, indicating that attention should be paid to the development of PSP even after the acute phase of stroke has passed. Only two sputum samples (8.3%) from patients with PSP were considered Geckler 4 or 5. In the present study, specimens were collected in an air-exposed environment such that facultative anaerobes, including *Escherichia coli* or *K. pneumoniae*, were cultured, whereas obligate anaerobes were not cultured. Aspiration is a leading cause of PSP [3,8,9], and obligate anaerobes, such as *Prevotella* spp., *Fusobacterium* spp., or *Bacteroides* spp., were occasionally detected from bronchoalveolar lavage fluid of patients with aspiration pneumonia [23]. The results of this study suggest that it is difficult to determine antimicrobial agents for PSP based on the results of sputum culture because uncultured organisms may be the initiating organisms, and it was difficult to obtain good-quality sputum samples of Geckler 4 or 5.

As initial treatment for PSP, broad-spectrum antimicrobial agents capable of covering anaerobes, such as ABPC/SBT and TAZ/PIPC, were administered. Of the three patients who died in the PSP group, pneumonia was the direct cause of death in only one case, and the other patient with PSP improved, suggesting that these antimicrobial choices were appropriate. Prophylactic antimicrobial therapy reduced infections; however, it did not improve prognosis [14]. Multiple antimicrobials were administered in cases of repeated PSP or prolonged antimicrobial therapy because of suspicion of infection at other sites. One case of PSP-related death was complicated by candidiasis; therefore, it is necessary to terminate antimicrobial therapy at the appropriate time.

A study has indicated that NGT insertion is a risk factor for PSP [1]. In the present study, 7 (33.3%) and 12 (4.7%) patients in the PSP and non-PSP groups, respectively, were inserted with NGT upon discharge. NGT insertion was performed during the course of admission in 16 of the patients with PSP, of whom 11 had an NGT inserted after PSP onset. Since NGT was inserted for the treatment of PSP, whether NGT insertion was a risk factor for PSP was unclear in this study.

ACE-I [24,25,26,27] and cilostazol [28,29] have been identified as drugs with prophylactic effects for PSP. In this study, ACE-I was used more frequently in the PSP group at the time of hospital discharge. However, all patients in the PSP group who received ACE-I were started following PSP onset, making the prophylactic effect of ACE-I on PSP unclear. Regarding cilostazol, no significant difference in the rate of use was noted in the PSP and non-PSP groups. Cilostazol was administered at the discretion of the attending physician when the cause of cerebral infarction was LA or SV. Owing to the side effect of bleeding, the use of cilostazol for preventing PSP was not recommended [30]. In practice, cases of SV were less likely to be the cause of AIS in PSP, and cilostazol is less likely to be used in cases of CE. The preventive effect of cilostazol on PSP needs to be compared among the same stroke subtypes.

This study had some limitations. First, it was a relatively small, single-center, retrospective study. Therefore, multivariate analysis to identify predictors of PSP development was impossible. Second, we examined the complication rate of supra- and infratentorial lesions in patients with PSP. However, we did not compare carotid versus vertebrobasilar arterial lesion. The comparison of PSP incidence with cerebral vascular territory of infarction and the examination of cerebral blood flow by single-photon emission computed tomography are agendas for future study. Third, ABPC/SBT was often the antimicrobial of choice for PSP. However, it was unclear whether a less expensive, narrow-spectrum antimicrobial agent would have been better. Furthermore, it was unclear whether NGT insertion was appropriate in the PSP group. Lastly, it was impractical to call in speech-language pathologists, otolaryngologists, or other specialists who could properly evaluate dysphagia immediately after PSP onset. It is necessary to consult with specialists in swallowing evaluation to determine which PSP cases are appropriate for NGT insertion.

## 5. Conclusions

We report the results of the clinical examination and treatment of patients with AIS who developed PSP. PSP was a poor prognostic factor for AIS. Although the ISAN score may be useful for predicting PSP, 37.5% of the patients with PSP were in the ISAN score low-risk group, indicating that accurate prediction was challenging. This study revealed that accurately identifying the causative organisms of PSP was difficult and that antimicrobial agents, including ABPC/SBT and TAZ/PIPC, NGT insertion, and ACE-I tended to be used more frequently in PSP cases. However, the definitive treatment of PSP cases needs to be studied in detail in a large number of cases.

## Figures and Tables

**Table 1:** Characteristics of PSP and non-PSP groups.

	PSP *n* = 24	Non-PSP *n* = 257	Total *n* = 281	*p* Value
Age	77 (65–94)	73 (39–99)	73 (39–99)	0.060
Male	17 (70.8%)	172 (66.9%)	199 (72.4%)	0.822
Independent before stroke	17 (70.8%)	228 (88.7%)	245 (89.1%)	0.021 *
Past medical history				
Hypertension	20 (83.3%)	160 (62.3%)	180 (65.5%)	0.045 *
Diabetes mellitus	9 (37.5%)	71 (27.6%)	80 (29.1%)	0.345
Smoking habit	15 (62.5%)	147 (57.2%)	162 (58.9%)	0.671
Stroke	4 (16.7%)	57 (22.2%)	61 (22.2%)	0.371
Neurological findings				
GCS	14 (9–15)	15 (5–15)	29 (10.5%)	<0.001 *
Hemiparesis	12 (50.0%)	133 (51.8%)	145 (52.7%)	1.000
Dysarthria	14 (58.3%)	95 (37.0%)	109 (39.6%)	0.049 *
Facial weakness	9 (37.5%)	58 (22.6%)	67 (24.4%)	0.130
NIHSS	4 (0–18)	2 (0–34)	2 (0–34)	0.007 *
Types of ischemic stroke				
Large artery	1 (4.2%)	30 (11.7%)	31 (11.3%)	0.331
Cardio embolism	8 (33.3%)	65 (25.3%)	73 (26.5%)	
Small vessel	2 (8.3%)	62 (24.1%)	64 (23.3%)	
Other determined	4 (16.7%)	17 (6.6%)	21 (7.6%)	
Two or more causes	0 (0%)	17 (6.6%)	17 (6.2%)	
Negative evaluation	9 (37.5%)	66 (25.7%)	75 (27.3%)	
Lesion location **				
Supratentorial	19 (79.2%)	199 (77.4%)	218 (77.6%)	0.540
Infratentorial	10 (41.7%)	59 (23.0%)	69 (24.6%)	0.042 *
ISAN score	8 (4–15)	5 (0–20)	13 (4.7%)	<0.001
Risk group				
Low risk (0–5)	9 (37.5%)	156 (60.7%)	165 (60%)	0.006 *
Medium risk (6–10)	7 (29.2%)	75 (29.2%)	82 (29.8%)	
High risk (11–14)	7 (29.2%)	22 (8.6%)	29 (10.5%)	
Very high risk (15-)	1 (4.2%)	4 (1.6%)	5 (1.8%)	
Complication of UTI	4 (16.7%)	26 (10.1%)	30 (10.7%)	0.244
Prescription at discharge				
Cilostazol	2 (8.3%)	36 (14.0%)	38 (13.8%)	0.341
ACE-I	4 (16.7%)	13 (5.1%)	17 (6.2%)	0.046 *
Outcome at discharge				
mRS 0	64 (24.9%)	2 (8.3%)	66	<0.001
1	73 (28.4%)	3 (12.5%)	76	
2	41 (16%)	1 (4.2%)	42	
3	28 (10.9%)	4 (16.7%)	32	
4	35 (13.6%)	2 (8.3%)	37	
5	13 (5.1%)	9 (37.5%)	22	
6	3 (1.2%)	3 (12.5%)	6	
mRS > 3	18 (75.0%)	78 (30.3%)	96 (34.1%)	<0.001 *
Duration of admission	46 (9–181)	20 (5–145)	23 (5–181)	<0.001 *
Meal at discharge (survival cases)				
Normal meal	4 (19.0%)	180 (70.9%)	184 (66.9%)	<0.001 *
Soft meal	7 (33.3%)	58 (22.8%)	65 (23.6%)	0.341
Tube feeding (nasogastric tube)	7 (33.3%)	12 (4.7%)	19 (6.9%)	0.046 *
Tube feeding (gastric fistula)	0 (0%)	1 (0.4%)	1 (0.4%)	<0.001 *
Intravenous hyperalimentation	3 (14.3%)	3 (1.2%)	6 (2.2%)	<0.001 *

The data for the continuous variables are presented as median (range). *: *p* value < 0.05; **: Cases with both supra- and infratentorial lesions were counted in both; ACE-I: angiotensin-converting enzyme inhibitor; GCS: Glasgow coma scale; ISAN score: integer-based pneumonia risk score; mRS: modified Rankin scale; NIHSS: National Institutes of Health Stroke Scale; PSP: post-stroke pneumonia; UTI: urinary tract infection.

**Table 2:** Comparison between patients with good and poor outcome.

	Good Outcome (mRS < 3) *n* = 184	Poor Outcome (mRS ≥ 3) *n* = 97	*p* Value
Age	71 (39–95)	79 (46–99)	<0.001 *
Male	135 (73.4%)	54 (55.7%)	0.002 *
Independent before stroke	184 (100%)	61 (62.9%)	<0.001 *
Past medical history			
Hypertension	117 (63.2%)	63 (65.6%)	0.793
Diabetes mellitus	57 (31.0%)	23 (23.7%)	0.126
Smoking habit	113 (61.4%)	49 (50.5%)	0.052
Stroke	40 (21.6%)	21 (21.9%)	0.961
Neurological findings			
GCS	15 (13–15)	15 (5–15)	<0.001
Hemiparesis	71 (38.6%)	74 (76.3%)	<0.001 *
Dysarthria	62 (33.7%)	47 (48.5%)	0.011 *
Facial weakness	35 (19.0%)	32 (33.0%)	0.007 *
NIHSS	1 (0–10)	4 (0–34)	<0.001 *
Types of ischemic stroke			
Large artery	14 (7.6%)	17 (17.5%)	0.002 *
Cardio embolism	45 (24.5%)	28 (28.9%)	
Small vessel	52 (28.3%)	12 (12.4%)	
Other determined	15 (8.2%)	6 (6.2%)	
Two or more causes	7 (3.8%)	10 (10.3%)	
Negative evaluation	52 (28.3%)	23 (23.7%)	
Complication of UTI	9 (4.9%)	21 (21.6%)	<0.001 *
Outcome at discharge			
Duration of hospitalization	16 (5–104)	37 (9–181)	<0.001 *
Meal at discharge			
Normal meal	159 (86.4%)	24 (24.7%)	<0.001 *
Soft meal	24 (13%)	41 (42.3%)	
Tube feeding (nasogastric tube)	0 (0%)	19 (19.6%)	
Tube feeding (gastric fistula)	0 (0%)	1 (1%)	
Intravenous hyperalimentation	0 (0%)	6 (6.2%)	

The data for the continuous variables are presented as median (range). *: *p* value < 0.05; ACE-I: angiotensin-converting enzyme inhibitor; GCS: Glasgow coma scale; ISAN score: integer-based pneumonia risk score; mRS: modified Rankin scale; NIHSS: National Institutes of Health Stroke Scale; PSP: post-stroke pneumonia; UTI: urinary tract infection.

**Table 3:** Multivariable predictors of outcome.

Variables	*p* Value	aOR (95% CI)
PSP	0.002	6.903 (2.080–22.915)
NIHSS	<0.001	1.885 (1.563–2.273)
Sex (Female)	<0.001	2.876 (1.473–5.613)

Adjusted by age, NIHSS, PSP, and sex (female). aOR: adjusted odds ratio; NIHSS: National Institutes of Health Stroke Scale; PSP: post-stroke pneumonia; UTI: urinary tract infection; CI: confidence interval.

## Data Availability

The data that support the findings of this study are available on reasonable request from the corresponding author.

## Supplementary Materials

The following supporting information can be downloaded at: https://www.mdpi.com/article/10.3390/neurolint15010006/s1, Table S1: Sputum culture results of 24 patients with PSP.
