# Supplementary material for: Post-Stroke Pneumonia in Real-World Practice: Background, Microbiological Examination, and Treatment
Source: Neurol Int. 2023 Jan 9;15(1):69–77. doi: 10.3390/neurolint15010006 (PMC9844281; doi:10.3390/neurolint15010006)
Supplement: Supplementary file 1 [file neurolint-15-00006-s001.zip › neurolint-2121247-supplementary.pdf]

Supplementary Materials

Post-stroke pneumonia in real-world practice: background, microbiological examination, and treatment

Takayoshi Akimoto, Makoto Hara, Masaki Ishihara, Katsuhiko Ogawa, and Hideto Nakajima

List of Contents

1. Suppemental Table:

Table S1. Sputum culture results of 24 patients with PSP

Geckler group classification	Culture results	Number
Geckler 5<br>BSE<10, WBC>25 / field	*Enterobacter aerogenes*	1
Geckler 4<br>BSE<10-15, WBC>25 / field	*Klebsiella pneumoniae*	1
	MSSA, Streptococcus agalactiae	1
Geckler 3<br>BSE>25, WBC>25 / field	*Providencia stuartii, Pseudomonas aeruginosa, Proteus mirabilis*	1
	Bacillus cereus	1
	Escherichia coli	1
	Klebsiella pneumoniae, MSSA	1
	Streptococcus agalactiae, Escherichia coli	1
	Pseudomonas aeruginosa	1
	Klebsiella pneumoniae, Serratia marcescens	1
	Haemophilus influenzae	1
	Streptococcus equisimilis, Serratia marcescens, MRSA	1
	Klebsiella spp.	1
	MSSA	1
	Stenotrophomonas maltophilia	1
	Klebsiella pneumoniae, MSSA	1
	Enterobacter cloacae, Stenotrophomonas maltophilia	1
	MSSA, *Klebsiella pneumoniae, Streptococcus agalactiae*	1
Not detected or Geckler ≤ 2		6

BSE: buccal squamous epithelial; MSSA: Methicillin-sensitive *Staphylococcus aureus*; Methicillin-resistant *Staphylococcus aureus*; WBC: white blood cells
